# Electrochemical cell lysis of gram-positive and gram-negative bacteria: DNA extraction from environmental water samples

**DOI:** 10.1016/j.electacta.2020.135864

**Published:** 2020-04-01

**Authors:** Siwen Wang, Yanzhe Zhu, Yang Yang, Jing Li, Michael R. Hoffmann

**Affiliations:** Linde+Robinson Laboratories, California Institute of Technology, Pasadena, CA 91125, USA

**Keywords:** Electrochemical cell lysis (ECL), DNA extraction, Gram-positive and gram-negative bacteria, Environmental water samples

## Abstract

Cell lysis is an essential step for the nucleic acid-based surveillance of bacteriological water quality. Recently, electrochemical cell lysis (ECL), which is based on the local generation of hydroxide at a cathode surface, has been reported to be a rapid and reagent-free method for cell lysis. Herein, we describe the development of a milliliter-output ECL device and its performance characterization with respect to the DNA extraction efficiency for gram-negative bacteria (*Escherichia coli* and *Salmonella* Typhi) and gram-positive bacteria (*Enterococcus durans* and *Bacillus subtilis*). Both gram-negative and gram-positive bacteria were successfully lysed within a short but optimal duration of 1 min at a low voltage of ∼5 V. The ECL method described herein, is demonstrated to be applicable to various environmental water sample types, including pond water, treated wastewater, and untreated wastewater with DNA extraction efficiencies similar to a commercial DNA extraction kit. The ECL system outperformed homogeneous chemical lysis in terms of reaction times and DNA extraction efficiencies, due in part to the high pH generated at the cathode surface, which was predicted by simulations of the hydroxide transport in the cathodic chamber. Our work indicates that the ECL method for DNA extraction is rapid, simplified and low-cost with no need for complex instrumentation. It has demonstrable potential as a prelude to PCR analyses of waterborne bacteria in the field, especially for the gram-negative ones.

## Highlights

• An electrochemical cell lysis (ECL) device was developed based on local high pH at cathode surface.
• Different bacteria were successfully lysed with an optimal duration of 1 min at a low voltage of ∼5 V.
• Various environmental water samples were demonstrated with high DNA extraction efficiencies.

## Graphical abstract

An electrochemical lysis device has been developed for DNA extraction of gram-positive and gram-negative bacteria from varied environmental samples.

**Figure:**
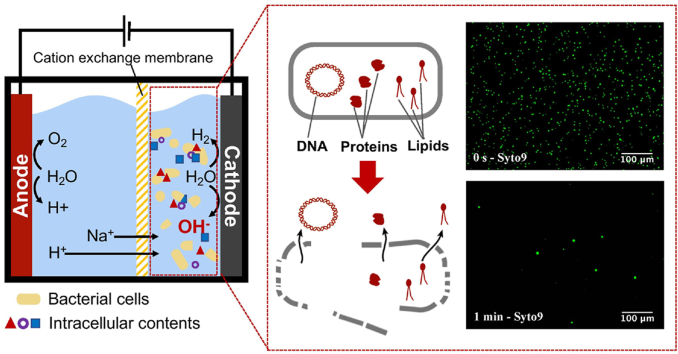

## 1 Introduction

During water electrolysis, the micro-environment at the electrode/electrolyte interface has different properties compare to that of the bulk electrolyte. The cathodic proton reduction to hydrogen significantly increases the pH at the surface of cathode. This mechanism plays important roles in various physio-chemical processes such as NH_3_ stripping [1], phosphate recovery [2] and enhanced CO_2_ reduction [3]. However, the application of this mechanism in biomolecular analysis, especially the detection of waterborne bacteria was relatively less explored.

In recent years, the application of biomolecular techniques such as polymerase chain reaction (PCR) has resulted in rapid, accurate, and sensitive methods for the quantification of waterborne bacteria [[4], [5], [6]]. The initial step before actual PCR analysis is cell lysis for the extraction of nucleic acids. One of the most common cell lysis technique for microbial quantification is chemical lysis, which employs an alkaline buffer or other lytic reagents to disrupt cell walls. This technique requires an array of essential instruments and multi-step reagent additions which are time-consuming and labor-intensive. In addition, removal of the reagents after cell lysis is required in order to avoid interference with downstream detection [7,8]. Electroporation uses the sharp potential gradient to break down cell membrane. It is fast and agent-free, and it is able to leave intracellular components intact [[9], [10], [11], [12], [13], [14], [15], [16], [17], [18]]. The downside of electroporation, however, is the use of high electric fields to achieve irreversible electroporation (*e.g.*, 10 kV/cm [14]). High power and voltage required to generate the high electric field, also leads to joule heating of the fluid [14,[19], [20], [21], [22]]. Lower electroporation voltages can be realized using nano-structured electrodes coupled with microfluidic devices. However, this approach would require a complicated fabrication process and precise operation [11,[13], [14], [15],^17^,[23], [24], [25]].

Electrochemical cell lysis (ECL) relies on the cathodically generated hydroxide (*i.e.*, localized high pH) to disrupt microbial cell membranes by breaking fatty acid-glycerol ester bonds in phospholipids [7,26]. In contrast to high-voltage electroporation (*e.g.*, 500 V [27]), ECL requires significantly lower voltages (*e.g.*, 2–5 V [7,8,26,28,29]), which avoids joule heating, and thereby, can be easily applied under resource limited conditions encountered in remote field sampling locations. However, we note that the aforementioned studies of ECL were mainly focused on clinical samples (*e.g.*, human cells [7,26]), and conducted in well-controlled systems with purified buffers. Furthermore, all of these studies highlighted in the development of micro-scale devices with microliter or even nanoliter throughput. It is important to understand if ECL can be used for other target cells with more common throughput that are related to more extensive applications, *e.g.,* environment, food and agriculture, *etc*.

Herein, we now report on the development and application of an ECL device that functions using a small sample volume (∼1 mL). Our overarching goal is to determine the DNA extraction efficiencies as a function of the key operational parameters (*i.e.*, pH ranges with varied treatment durations) for the use of ECL, as applied to DNA extraction and PCR amplification of gram-positive and gram-negative bacteria in real surface water and wastewater.

## 2 Experimental

### 2.1 Reagents

Sodium sulfate (Na_2_SO_4_) was purchased from EMD Millipore Corporation (Germany). Hydrochloric acid (HCl) and sodium hydroxide (NaOH) were purchased from Sigma-Aldrich (USA). 50 mM Na_2_SO_4_, HCl with varied concentrations (0.1 mM, 1 mM, 10 mM, 100 mM and 1 M) and NaOH with varied concentrations (0.1 mM, 1 mM, 10 mM, 100 mM and 1 M) were prepared using ≥ 18 MΩ Milli-Q water produced from a Millipore system (Millipore Co., USA). PBS (Gibco™, 1×, pH 7.2) was purchased from Thermo Fisher Scientific (USA). Luria-Bertani (LB) Broth, Tryptic Soy Broth (TSB), Brain Heart Infusion (BHI) Broth and Nutrient Broth (NB) were purchased from Becton, Dickinson and Company (USA). Nuclease free water for PCR was purchased from Promega Corporation (USA).

### 2.2 Bacterial sample preparation

The gram-negative bacteria species, *Escherichia coli* (ATCC 10798, *E. coli*), *Salmonella* Typhi (ATCC CVD909, *S.* Typhi), and gram-positive bacteria species, *Bacillus subtilis* (ATCC 6051, *B. subtilis*) and *Enterococcus durans* (ATCC 6056, *E. durans*) were cultivated at 200 rpm (Innova 42 Incubator Shaker, New Brunswick Scientific, USA) for 12–14 h to log-phase growth at the optical density at λ = 600 nm (OD_600_) of 0.6–1.0. *E. coli, S.* Typhi and *E. durans* were grown at 37 °C in LB, TSB and BHI media, respectively. *B. subtilis* was grown at 30 °C in NB media. After incubation, the bacterial cells were harvested by centrifugation (Eppendorf, Germany) at 5000 rpm, washed twice and resuspended in 50 mM Na_2_SO_4_ to a concentration of ∼10^8^ cells/mL (estimated by OD_600_ values).

### 2.3 Electrochemical cell lysis experiment

**Fig. 1:**
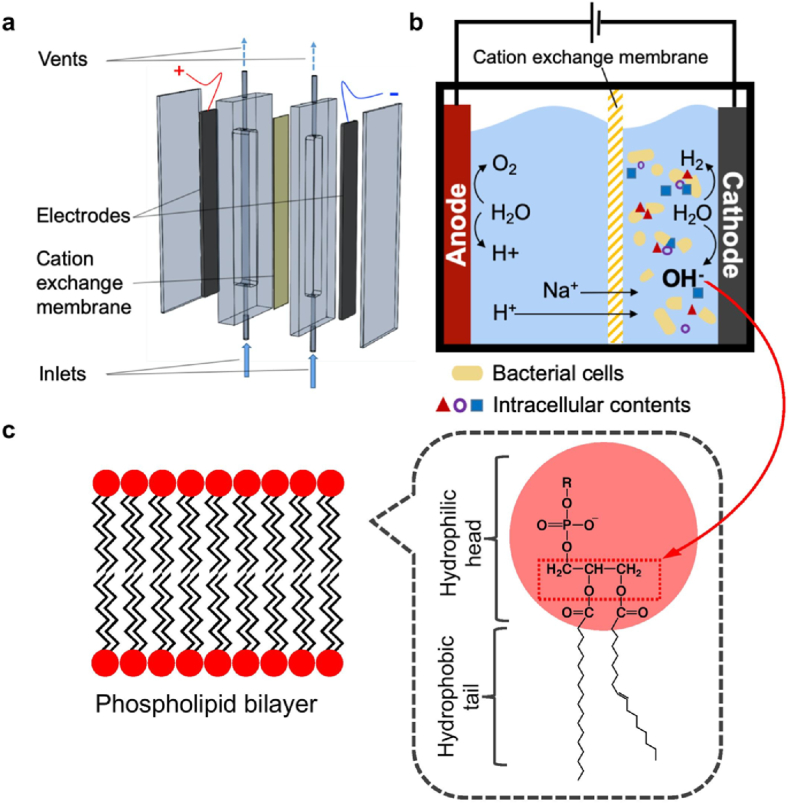
Device and mechanism of electrochemical cell lysis. (a) Electrochemical cell lysis device. (b) Schematics of electrochemical cell lysis with cation exchange membrane between anodic and cathodic chambers. (c) Phospholipid bilayer, the major component of bacterial cell membranes, and the chemical structure of phospholipids. The fatty acid-glycerol ester bonds in phospholipids (highlighted in red box) can be hydrolyzed by the locally generated OH^−^ at cathode. (For interpretation of the references to colour in this figure legend, the reader is referred to the Web version of this article.)

The ECL device consists of a dimensionally stable IrO_2_/Ti anode (synthesis was reported previously [30]), a Ti cathode, and a cation exchange membrane (Nafion 117, Dupont, USA), as shown in Fig. 1a. This is a typical configuration for water electrolysis. The reactor was made of polycarbonate and a photograph of the ECL device is also shown in Fig. S1. The mechanism on the breakdown of microbial cell membrane by ECL is illustrated in Fig. 1b and c. The membrane separates the device into an anodic chamber (1.6 mL) and a cathodic chamber (0.8 mL). One outlet was added on the top of each chamber to enable gas ventilation. For ECL reactions, 50 mM Na_2_SO_4_ and bacterial suspensions were injected from the bottom into the anodic and cathodic chamber, respectively, using syringes. A constant direct current of 40 mA (16 mA/cm^2^, Potentiostat, BioLogic Science Instruments, France) was applied for 30 s −10 min. The cathodic effluents were collected, using syringes, after each reaction and the chambers were washed three times with DI water between each reaction. The pH values were measured for all cathodic effluents and initial samples with a pH meter (Orion Star A215, Thermo Fisher Scientific, USA) containing a semi-micro pH probe (Orion 9110DJWP, Thermo Fisher Scientific, USA).

### 2.4 Analysis of cell lysis by fluorescent microscope

Following ECL reaction, a 500 μL aliquot of each bacterial sample was harvested by centrifugation at 10,000g for 10 min at 20 °C. The resulting pellets were then washed with PBS three times and resuspended in PBS to a final volume of 500 μL. The Live/Dead Baclight Viability kit (Invitrogen by Thermo Fisher Scientific, USA) was used for bacterial staining. Two staining dyes are included in this kit, the green-fluorescent nucleic acid stain Syto9, which stains both live and dead cells, and the red-fluorescent nucleic acid stain propidium iodide (PI), which can penetrate and stain only dead cells due to their compromised membrane [31]. The viability of bacterial cells was monitored by these two dyes. PI-staining of dead cells does not indicate the complete rupture of cell membranes, but merely their permeability for PI. Since completely lysed cells cannot be stained by Syto9, the extent of cellular lysis was measured by counting cells stained by Syto9 before and after ECL, as shown in Eq. (1) below:

(1) $$Lysis\;efficiency\;\left(\%\right)=\frac{N_{total\;cells\;in\;initial\;sample}-N_{total\;cells\;in\;ECL\;sample}}{N_{total\;cells\;in\;initial\;sample}}\times 100$$

where *N* is the counted number of the cells that stained by Syto9. According to the manufacturer’s instruction, equal volumes (1.5 μL) of Syto9 (0.33 mM) and PI (2 mM) were added into each 100 μL sample. Each stained sample was added onto a glass slide with cover and examined under a fluorescence microscope (Leica DMi8, Germany). An objective with × 20 magnification was used for analyses. Five images were randomly taken from different areas on each slide and counted by ImageJ software (National Institute of Health, USA).

### 2.5 DNA quantification by qPCR

To measure the DNA released by ECL, the suspended DNA was collected from the supernatant of each sample by centrifugation at 10,000g for 10 min. As a negative control, an aliquot of the initial sample without ECL was treated in the same way to remove all the cells. Another aliquot of the initial sample was extracted for each bacterial strain using a commercial DNA extraction kit (PureLink® Genomic DNA Mini Kit, Invitrogen by Thermo Fisher Scientific, USA) as a positive control. Real-time PCR (qPCR, MasterCycler RealPlex 4, Eppendorf, USA) was used to quantify the presence of the universal bacterial 16S rRNA gene and to analyze DNA extraction efficiency for all the above samples. Each sample was tested in triplicates, using a similar protocol as reported previously [3,32]. The protocol was also briefly described in the Supporting Information, along with other necessary information for qPCR quantification including amplification curves (Fig. S2), qPCR standard curves and PCR efficiencies (Fig. S3). The cycle numbers above the background fluorescence threshold (C_T_) were directly measured and analyzed after PCR reaction, using instrument specific software (Eppendorf, USA). The higher the DNA concentration in the template, the lower the C_T_ value because the background threshold can be reached with less cycles of PCR amplification. To evaluate the DNA extraction efficiency, ΔC_T_ values of the ECL treated samples were calculated by subtracting C_T_ values of the suspended DNA in the ECL treated samples from those in the untreated ones. With a comparison, ΔC_T_ values of the samples extracted by the commercial kit were calculated similarly, by subtracting C_T_ values of the total DNA extracted by the commercial kit from those of the suspended DNA in the untreated samples. For each bacterial strain, the higher ΔC_T_ values were expected for higher DNA extraction efficiency.

### 2.6 pH effect tests

The investigation of pH effects on cell lysis was conducted for one gram-negative bacterial species (*E. coli*) and one gram-positive species (*E. durans*) without ECL reaction. *E. coli* and *E. durans* were cultivated as described above. Then, several aliquots of 1 mL bacteria suspensions were harvested by centrifugation at 5000 rpm to obtain pellets. After removal of the culture media, 500 μL of NaOH with different concentrations (0.1 mM, 1 mM, 10 mM, 100 mM and 1 M) were directly added to the cell pellets, respectively, and resuspended immediately. As a negative control, 1 mL of 50 mM Na_2_SO_4_ was added to the cell pellets of initial samples for both species and mixed well. 500 μL of HCl with varied concentrations (0.1 mM, 1 mM, 10 mM, 100 mM and 1 M) were then added to neutralize the alkaline samples, correspondingly, after different sample contact times with alkaline solution (30 s, 1 min, 2 min, 5 min and 10 min). All the neutralized samples were centrifuged at 10,000g for 10 min to remove all the intact cells. The supernatants were then purified by the PureLink® Genomic DNA Mini Kit. An aliquot of the control was extracted by the same commercial DNA extraction kit as a comparison. Another aliquot was treated the same as other samples after alkaline lysis. Then all the purified samples were quantified by qPCR and ΔC_T_ values were calculated with the same methods described in the section of DNA quantification by qPCR.

### 2.7 Electrochemical cell lysis of bacteria in environmental water samples

Three different environmental water samples were tested to evaluate the performance of the ECL technique on DNA extraction of bacteria from ambient environmental water. Pond water was collected from the turtle pond at Caltech campus (Pasadena, CA). The treated and untreated latrine wastewater was collected from a previously described solar-powered recycling electrochemical toilet system located at Caltech with 550 mg/L of chemical oxygen demand (COD) and 28 mM NH_4_^+^ as major pollutants [33,34]. The latrine wastewater was treated by an electrochemical oxidation process to remove >90% of NH_4_^+^ and COD. Effluent was collected and denoted as “treated water” in this study. Pond water was directly added into the cathodic chamber for ECL reaction, without any pretreatment while 50 mM Na_2_SO_4_ was added into the anodic chamber. Both types of wastewater samples were first filtered, using sterilized filter papers with 8.0 μm pore size (diameter, 55 mm; Cat No., 1002 055; Whatman) to remove big particles and to enhance the reproducibility between each experiment. Then the filtered wastewater was added into cathodic chamber for ECL reaction while 50 mM Na_2_SO_4_ was added into the anodic chamber. The suspended DNA of total bacteria from all the environmental water samples were then collected by centrifugation at 10,000g for 10 min. All the above environmental water samples were also extracted by the same commercial DNA extraction kit (PureLink® Genomic DNA Mini Kit) as the positive control. The same qPCR method was used for DNA quantification and evaluation of DNA extraction efficiency.

## 3 Theory and simulations

COMSOL Multiphysics (COMSOL Inc., USA), a commercial finite element modeling software, was used to study the fate and transport of hydroxide ions inside the cathodic chamber. The fluid in the cathodic chamber was modeled as a 3 × 5 × 50 mm^3^ block, with the electrode surface and the cation exchange membrane represented by the two 5 × 50 mm^2^ sides. The gas vent hole on the top was represented by a cylindrical extrusion with a diameter of 1 mm and a height of 0.1 mm. OH^−^ and H^+^ are generated with the hydrogen and oxygen evolution reactions at the cathode and anode, respectively:

(2) $$\text{anode:}\;2H_{2}O↔4H^{+}+4e^{-}+O_{2}$$

(3) $$\text{cathode:}\;4H_{2}O+4e^{-}↔4OH^{-}+2H_{2}$$

The generation and venting of H_2_ during electrolysis induces fluid movements in the cathodic chamber. The resulting flow field was first calculated and then, the convective and diffusive OH^−^ transport was simulated. Molar influx of H_2_ gas at the cathode surface was theoretically half of the OH^−^ generation rate $R_{in}^{cat}$, which was calculated by Ref. [35]:

(4) $$R_{in}^{cat}=\frac{i}{nFA}$$

where i is the supplied current (40 mA), *n* is the number of electrons used to generate a hydroxide ion, which is 1, *F* is the Faraday constant, and *A* is the surface area. Simultaneously, H^+^ was produced at the anode surface at the same rate as OH^−^ was generated, and cations were forced across the cation exchange membrane. It was assumed that sodium ions were the dominant species transported across the membrane due to their concentration dominance over protons, until sodium ions were depleted to a concentration comparable to the protons; at this point protons were the preferred ions for membrane transport due to their smaller size. For the cathodic chamber, the influx of H^+^ was considered as the sink of OH^−^ and the contribution of water dissociation was negligible to mass transfer trough the membrane [[36], [37], [38]]. With the initial pH set at 7.5, time-dependent OH^−^ concentration profiles were simulated over the whole geometry. The transient pH profiles of the vertical mid-plane across the electrode and the membrane were generated, while the bulk solution pH was estimated from the volume average of [OH^−^]. More details on the modules and equations used in this simulation are shown in the Supporting Information.

## 4 Results and discussion

### 4.1 Electrochemical cell lysis of different bacteria

**Fig. 2:**
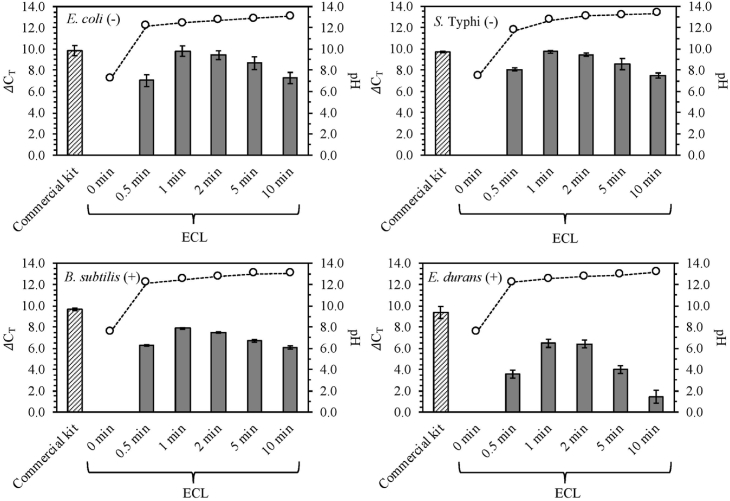
ΔC_T_ values of 4 different bacterial cells lysed by ECL as a function of times () and of those extracted by a commercial DNA extraction kit () as a comparison; and the average pH values measured in the cathodic effluents (). For the ECL treated samples, ΔC_T_ values were calculated by subtracting C_T_ values of the suspended DNA in ECL treated samples from those in the untreated samples. For the samples extracted by the commercial kit, ΔC_T_ values were calculated by subtracting C_T_ values of the total DNA extracted by the commercial kit from those of the suspended DNA in the untreated samples.

Four different bacteria, *E. coli*, *S.* Typhi, *B. subtilis* and *E. durans*, with the initial concentrations of approximately 10^8^ cells/mL were effectively lysed using the ECL method at different durations. ΔC_T_ values of 4 different bacteria treated by ECL with 30 s-10 min are shown in Fig. 2, along with a comparison of those extracted by the commercial kit. After 30 s of ECL, the averaged ΔC_T_ values of all the bacterial strains were significantly increased to 3.6–8.1. The highest ΔC_T_ values of the ECL treated bacterial samples all lied in the duration of 1 min as the optimized ECL condition, with the range of 6.5–9.8. In general, the DNA extraction efficiencies of all the bacterial cells decreases after 2 min of ECL. This could be mainly due to DNA damage during ECL process (*e.g.*, the local high pH which will be further discussed later with simulation in this study), as we preclude PCR inhibition caused by electrolyzed cathodic effluents. The details are described in Supporting Information, Fig. S4 and Fig. S5. The pH of the catholyte increased rapidly from the average of 7.4 (±0.2) to 12.5 (±0.1) after 1 min of ECL, which is consistent with the increase of ΔC_T_ values. It confirms that the generation of OH^−^ at cathode is the mechanism of ECL. All the PCR mixtures containing cathodic effluents (after ECL) were able to be adjusted to a pH range of 8.4–8.7 by the PCR reagents prior to qPCR measurements. Thus no additional neutralization step was necessary before detection. The optimized ECL duration of 1 min is much faster than most of the commercial DNA extraction kits based on chemical lysis (*e.g.*, at least 30 min for lysis step with the PureLink® Genomic DNA Mini Kit) [39]. The optimal processing time by ECL is also faster than the typical processing time of 5–30 min by the bead beating method, when using a flat pad vortex mixer, which is the least expensive bead beating technique [40,41]. In addition, the required voltage input is *ca.* 5 V, which is ∼10–1000 fold lower than that of electrical lysis, reported previously [17,[42], [43], [44]].

DNA extraction by ECL was especially efficient for the 2 gram-negative bacterial strains. The averaged ΔC_T_ values increased to 9.8 and 9.7 with 1 min of ECL for *E. coli* and *S.* Typhi, respectively. There is no significant difference between the ΔC_T_ values of the samples treated by 1 min of ECL and of those extracted by the commercial kit (*P* = 0.72 for *E. coli* and *P* = 0.48 for *S.* Typhi). Lower DNA extraction efficiencies were observed for the 2 gram-positive bacterial strains with the optimized 1 min of ECL. Compared to the samples extracted by the commercial kit, the differences of ΔC_T_ values (= ΔC_T, commercial kit_ - ΔC_T, 1 min of ECL_) are 1.8 and 2.9 for *B. subtilis* and *E. durans*, respectively. However, the ΔC_T_ values after 1 min of ECL were still increased significantly to 7.9 and 6.5 for *B. subtilis* and *E. durans*, respectively, which was sufficient for downstream qPCR detection in this study. The lower lysis efficiency for gram-positive bacteria than for gram-negative bacteria was not only observed by using ECL in our present study, but also by other lysis methods reported previously. For example, a lysis method based on cold atmospheric-pressure plasma was reported to have only 0.6 log_10_ reduction for *B. subtilis* after 10 min treatment, while 3.3–3.6 log_10_ reduction for other 3 gram-negative bacteria with the same treatment duration [45]. And 10–100 times higher detection limits were determined for gram-positive bacteria than for gram-negative bacteria by applying a hybrid chemical/mechanical lysis method on a microfluidic chip [46]. The differences in DNA extraction efficiency between the gram-positive and gram-negative bacteria can be explained by their different cell wall structures. The cell walls of gram-negative bacteria are composed of phospholipid bilayers (*i.e.*, cell membranes) that can be readily hydrolyzed by hydroxide ions, while the cell walls of the gram-positive bacteria are predominantly composed of multilayers of peptidoglycan, which provide stronger protection for gram-positive bacteria [[47], [48], [49]]. In addition, the cell wall thickness of gram-positive bacteria (*e.g.*, ∼55.4 nm for *B. subtilis* [[50], [51], [52]]) is generally much higher than that of gram-negative bacteria (*e.g*., ∼8.2 nm for *Enterobacter cloacae* [53,54]) as well.

**Fig. 3:**
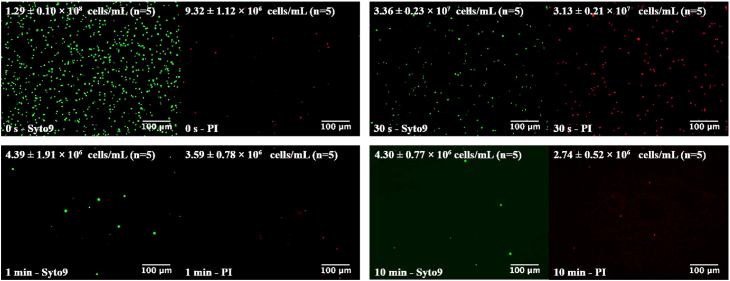
Fluorescent microscope images of *E. coli* cells stained by Syto9 (green) and PI (red) with different durations of ECL. (For interpretation of the references to colour in this figure legend, the reader is referred to the Web version of this article.)

The successful cell lysis by ECL was further confirmed for all the bacteria via fluorescence microscopy. The fluorescence images visualizing the bacteria viability with ECL treatment, monitored by PI (in red) and Syto9 (in green), are shown for *E. coli* as an example in Fig. 3. It was observed clearly that cells were completely lysed by ECL after the cell death. Because the number of dead cells (in red) significantly increased after only 30 s of ECL, but reduced after 1 min. So did most of the total intact cells (in green) disappear after 1 min, which is an evidence for complete cell wall breakdown. The images in fluorescent green also show that the number of total intact cells decreased significantly after 30 s of ECL and only a few can be observed after 1 min, which has an agreement with the increase of ΔC_T_ values measured by qPCR. The cell numbers for both live and dead cells were calculated for all the bacteria with different ECL durations and are shown in Fig. S6. For gram-negative bacteria, the lysis efficiencies are close to 100% after 2 min of ECL, while efficiencies over 50% for both of the gram-positive bacteria were obtained after 5 min. The lysis efficiencies keep increasing over time until an apparent equilibrium is achieved. Apparently, the cell number measurement by fluorescent microscope showed the efficient performance of ECL on cell lysis more straightforwardly, due to the absence of complex factors related to DNA detection, *e.g.*, potential DNA damage after release from cells and PCR inhibition.

### 4.2 pH effects on cell lysis and DNA extraction

**Fig. 4:**
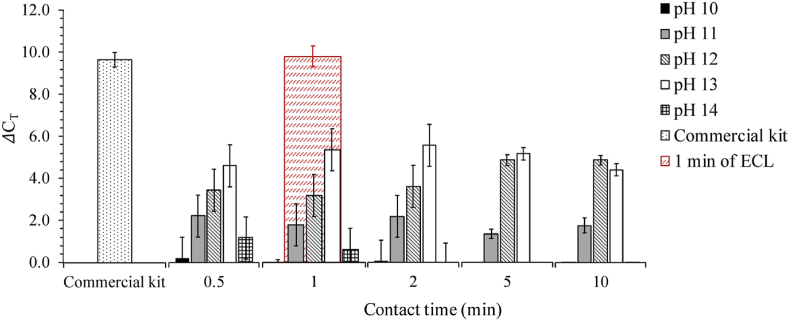
ΔC_T_ values of *E. coli* cells under varied pH conditions as a function of contact times, with comparison of those extracted by the commercial DNA extraction kit and 1 min of ECL.

To further understand how pH affects cell lysis and DNA extraction, bacterial cells were treated by homogeneous alkaline lytic reagent at various pH values, *i.e.*, NaOH with varied concentrations of 0.1 mM–1 M, without ECL. *E. coli* and *E. durans* were selected as models for gram-negative and gram-positive bacteria, respectively. Homogeneous alkaline lysis is not efficient for *E. durans* at all investigated pH values (10–14). The ΔC_T_ values of *E. durans* treated by NaOH were all lower than 3.0 (data not shown), while those extracted by the commercial kit were 11.6 as an average. The ΔC_T_ values of *E. coli* cells treated by NaOH at varied pH from 10 to 14 as a function of contact times, are shown in Fig. 4. *E. coli* cells were barely lysed at pH 10 with ΔC_T_ values close to 0, while higher DNA extraction efficiency was observed at pH 11 with ΔC_T_ values around 2. Among all the conditions, the highest DNA extraction efficiency for *E. coli* cells was achieved at pH 13 with an averaged ΔC_T_ value of 5.6 at 2 min contact time. However, ΔC_T_ values decreased at contact times longer than 2 min. When pH increased to an even higher level, *i.e.*, at pH 14, C_T_ values of NaOH treated cells were even lower than initial samples after 2 min of contact time, although the samples were neutralized after a defined contact time. Consequently, ΔC_T_ values were negative and cannot be seen in Fig. 4. This suggests that the DNA might be damaged by high pH conditions above pH 13, which has an agreement with the DNA damage observed in the ECL experiments with longer durations than 2 min. On the other hand, there was no decrease of ΔC_T_ values observed for NaOH treated *E. coli* cells at pH 12 within contact times of 10 min. The ΔC_T_ values at pH 12 were quite close to those at pH 13 after 5 min of contact time and even out performed those at pH 13 later on. Therefore, it appears that a pH between 12 and 13 may provide optimal conditions for DNA extraction from bacterial cells; this result is consistent with a previously reported optimal pH range of 11.5–12.5 for cell lysis [8,55]. Plasmid DNA isolation via alkaline lysis was also previously reported to be most efficient within a pH range of 12.0–12.6 [56,57]. These values are also in good agreement with the bulk pH (12.47–12.76) measured under optimized conditions during ECL extraction.

As a comparison, the highest averaged ΔC_T_ value achieved by alkaline lysis (pH 13, 2 min) is 4.2 less than of that measured after 1 min of ECL, as highlighted in Fig. 4. And *E. coli* cells extracted by the commercial kit in this pH test were detected as similar ΔC_T_ values (9.7 ± 0.3) to those treated by 1 min of ECL. Besides, ECL is also capable of lysing gram-positive bacteria while conventional alkaline lysis cannot. Although the released DNA could be damaged by the high pH raised with longer ECL duration, the lysis of bacterial cells could also be benefited from the local high pH generated at cathode during ECL process. These results emphasize that the ECL method is faster and much more efficient for DNA extraction from gram-negative and gram-positive bacterial cells, compared to alkaline cell lysis.

### 4.3 Simulations of pH profiles at the cathode

**Fig. 5:**
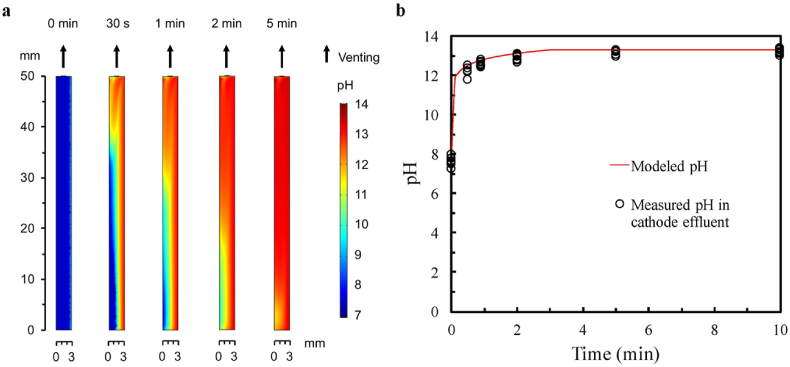
Computational simulation results for the distribution of pH in the cathodic ECL chamber and corresponding pH values of cathodic effluents. (a) Simulation of pH value distribution for the vertical mid-plane in the cathodic chamber with the cation exchange membrane on the left and the cathode on the right. (b) Modeled and measured pH for the cathode effluents as a function of electrochemical reaction time.

To gain more mechanistic insight of the ECL process, pH profiles for the vertical mid-plane of the cathodic chamber were simulated for different contact times and are shown in Fig. 5a. These simulations show that the local pH value near the cathode surface increases rapidly within 1 min of ECL and that an ideal pH range for cell lysis (pH 12–13) is predicted. After 2 min of ECL operation, the pH in most of the upper volume reaches 13. This simulation is consistent with the DNA loss observed during ECL tests on different bacteria. Hydrogen gas is also generated, as protons are consumed and OH^−^ is produced at the cathode surface. Gas evolution helps mixing the solution (the calculated flow field is shown in Fig. S7), which in turn leads to a larger volume that has a suitable pH for cell lysis after 30 s and 1 min of operation (Fig. 5a). The simulated pH profiles for the bulk-phase cathodic solutions as a function of time is shown in Fig. 5b. The simulation results are in line with the measured bulk pH values of the cathodic effluents during different ECL tests. The results also highlight that there is a higher pH at the cathode surface than in the bulk electrolyte. It is speculated that cells were efficiently lysed near the cathode surface as we discussed earlier in this study. The released DNA molecules with negative charge were likely repelled from the cathode, and subsequently preserved in the bulk electrolyte at a lower pH. This may explain the much more efficient DNA extraction by ECL than that by direct alkaline lysis, which was found in the pH effect tests (*vide supra*). Detailed understanding of this phenomenon awaits further study.

### 4.4 Electrochemical cell lysis in environmental water

**Fig. 6:**
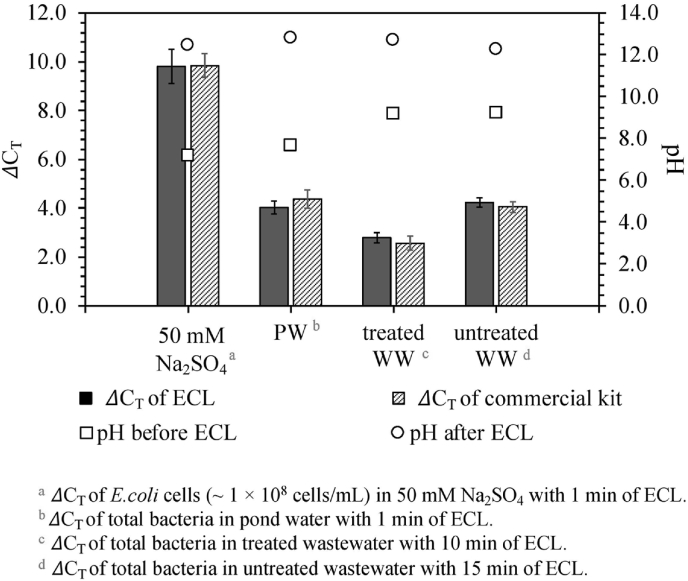
ΔC_T_ values of bacterial cells in 50 mM Na_2_SO_4_, pond water (PW), treated wastewater (treated WW) and untreated wastewater (untreated WW) extracted by ECL and the commercial kit.

Fig. 6 shows the optimal ΔC_T_ values of total bacteria in natural pond water, treated and untreated latrine wastewater treated by ECL, with the comparison of those of *E. coli* (∼10^8^ cells/mL) in 50 mM Na_2_SO_4_ treated by ECL (*vide supra*). The initial cell concentrations of total bacteria were approximately 8.0 × 10^5^, 3.0 × 10^6^, 2.1 × 10^7^ cells/mL for pond water, treated and untreated wastewater, respectively, as measured by qPCR with the calibration curve of *E. coli* (shown in Fig. S3). The optimal DNA extraction efficiency achieved ΔC_T_ values of 4.4 ± 0.4 for pond water after 1 min of ECL. For the treated and the untreated wastewater samples, the optimal ΔC_T_ values of 2.6 ± 0.3 and 4.1 ± 0.2 were obtained after 10 min and 15 min of ECL, respectively. These results show that the bacteria in both pond water and wastewater were rapidly and efficiently lysed by ECL with ΔC_T_ values comparable to those obtained with the commercial kit. The differences of ΔC_T_ values between ECL and the commercial kit are generally less than 0.3 for different water types. Clearly, the required lysis/extraction times for environmental water samples are longer than those for pure cell samples reported herein. It could be mainly taken account of the more complex composition in real environmental water samples which has buffer capacity. Therefore, it takes longer reaction time to achieve the ideal pH range for cell lysis in the cathodic chamber. For example, it was reported previously that there was 17 mM of HCO_3_^−^ + CO_3_^2−^, 0.6 mM of total phosphate and 13 mM of NH_4_^+^, with buffer capacity of 0.79, 0.09 and 2.71 mequiv/(L⋅ pH), respectively, for the wastewater collected from the same onsite electrochemical wastewater treatment system as this study [58]. However, the ECL process is still much faster than most of the conventional DNA extraction kits (*vide supra*), additionally with much more simplified operational procedure.

The optimized DNA extraction efficiencies for the environmental water samples by ECL treatment were in a pH range from 12 to 13. These results suggest that the pH can be used as an indicator to determine the optimal residence time of ECL for DNA extraction in the field. Additionally, in this study, a centrifugation step (at 10,000g for 10 min) was applied after each ECL reaction because the cell lysis by PCR process needs to be excluded for measuring the DNA extraction efficiency by ECL *per se*. The thermal cycling process of PCR could also cause some of the cells lysed and thereby increased the DNA extraction efficiency. Fig. S8 shows that the qPCR C_T_ values are 0.4–1.0 lower for different environmental water samples without any further treatment after the optimized ECL than with the centrifugation step. This result is somewhat counter-intuitive since higher C_T_ values (lower DNA concentrations) were expected for the samples without post-ECL treatment due to the potential inhibitors in environmental samples. However, any post-treatments after lysis could also cause sample loss, which might explain the lower C_T_ values (higher DNA concentrations) detected in this study. Therefore, for application of ECL in the field, the centrifugation after ECL might not be necessary. In case that a treatment might be necessary to reduce PCR inhibition, a filtration step with a 0.2 μm syringe filter (13 mm, nylon, Pall Corporation, USA) was also tested after ECL as an alternative post-treatment to centrifugation. Because it is much easier to be realized in the field. Centrifugation and filtration as a post-ECL step resulted in no significant differences of qPCR C_T_ values (*P* = 0.62, 0.25 and 0.48 for pond water, treated and untreated wastewater, respectively) for the three different types of environmental water samples (shown in Fig. S8).

## 5 Conclusion

In summary, we developed an ECL device for the rapid extraction of DNA from waterborne bacteria, using low-cost materials. The efficient cell lysis by ECL was demonstrated for both gram-positive and gram-negative bacteria with a short but optimal lysis duration of 1 min, at a constant DC of 40 mA (∼5 V of voltage). Extraction by ECL was more efficient and quicker than direct alkaline lysis. The successful application of ECL on different environmental water samples suggests the potential application of ECL as a rapid and reagent-free sample preparation technique with a low voltage requirement for microbial monitoring in the field. In addition, ECL as applied to cell lysis has the potential to significantly reduce the overall cost for nucleic acid-based microbial monitoring. For example, a conventional DNA extraction kit, based on chemical lysis, *e.g.*, PureLink® Genomic DNA Mini Kit, costs approximately $3 per preparation, using the required instrumentation (*e.g.*, centrifuge ($2000–20,000 provided by Eppendorf) and vortex mixer (>$300 available through VWR)). The bead beating method costs *ca.* $2 per sample prep using 0.1 mm diameter beads (Gene Rite, LLC) and a bead milling instrument with a price range from $300 to $12,000 [[59], [60], [61], [62], [63]]. The ECL device developed in this study, on the other hand, can be produced for as little as $4.20 per unit. The estimated total cost includes a) polycarbonate reactor ($0.44), b) an anode ($0.8 for an IrO_2_/Ti anode with an estimated lifetime of 4.3 yrs at 25 mA/cm^2^, as reported previously [30]), c) $0.54 for the Ti-cathode and, d) a cation exchange membrane ($2.42 for Nafion 117 with estimated lifetime of >60,000 h [64,65]). For field sampling, the ECL device can be powered by 4 AA batteries that should cost less than $1 for typical alkaline batteries.

## CRediT authorship contribution statement

**Siwen Wang:** Investigation, Data curation, Formal analysis, Writing - original draft. **Yanzhe Zhu:** Methodology, Software, Writing - original draft. **Yang Yang:** Conceptualization, Methodology, Writing - review & editing. **Jing Li:** Writing - review & editing. **Michael R. Hoffmann:** Supervision, Funding acquisition, Writing - review & editing.

## Declaration of competing interest

There are no conflicts of interest to declare.
