# Supplementary material for: Electrochemical cell lysis of gram-positive and gram-negative bacteria: DNA extraction from environmental water samples
Source: Electrochim Acta. 2020 Apr 1;338:135864. doi: 10.1016/j.electacta.2020.135864 (PMC7063685; doi:10.1016/j.electacta.2020.135864)
Supplement: Multimedia component 1 [file mmc1.docx]

*-Supporting Information-*

**Electrochemical cell lysis of gram-positive and gram-negative bacteria:**

**DNA extraction from environmental water samples**

Siwen Wang, Yanzhe Zhu, Yang Yang, Jing Li and Michael R. Hoffmann ^*^

Linde+Robinson Laboratories

California Institute of Technology

Pasadena, California 91125 USA

^*^Corresponding Author

Phone: +1 626-395-4391

Fax: +1 626-395-2940

E-mail: [mrh@caltech.edu](mailto:mrh@caltech.edu).

Submitted to *Electrochimica Acta*

February 2020

**
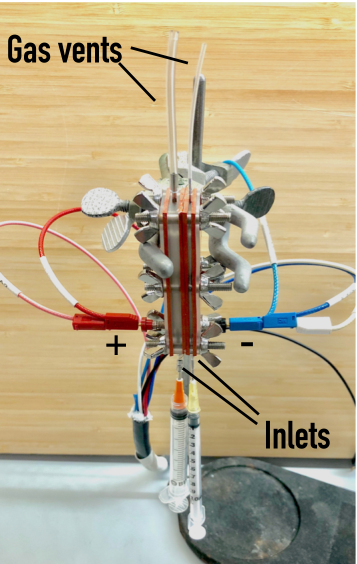
**

**Figure S1.** The photograph of electrochemical cell lysis (ECL) device.

**Detailed methods and information of qPCR measurements**

Real-time PCR (qPCR, MasterCycler RealPlex 4, Eppendorf, USA) was used to quantify the universal bacterial 16S rRNA gene. Each 20 µL reaction mixture contains 2 µL of sample, 10 μL of PerfeCTa® qPCR ToughMix® (Quanta BioSciences Inc.), 0.25 μM of both forward (1369F, 5′CGG TGA ATA CGT TCY CGG3′, where Y is either C or T, Integrated DNA Technologies Inc., USA) and reverse primers (1492R, 5′GGW TAC CTT GTT ACG ACT T3′, where W is either A or T, Integrated DNA Technologies Inc., USA) and 0.25 μM of TaqMan probe (FAM-5′CTT GTA CAC ACC GCC CGT C3′, Integrated DNA Technologies Inc., USA). The thermal cycling was 3 min at 95 ^o^C followed by 40 cycles of 15 s at 95 ^o^C for denaturation and 30 s at 55 ^o^C for annealing/extension. The PCR amplification curves are shown as an example in Figure S2. A non-template control (NTC) was set up with each qPCR test. The average C_T_ values for all the NTCs were 35.8 (± 0.50, n=9), which meets the requirement suggested by the EPA protocol “Method B: *Bacteroidales* in Water by TaqMan® Quantitative Polymerase Chain Reaction (qPCR) Assay” [1]. As a control test, 50 mM Na_2_SO_4_­ was injected into the cathodic chamber after three-times wash following an ECL experiment with bacterial cells. The effluent was collected after 10 min and detected by qPCR. The average qPCR C_T_ values are 32.0.

The qPCR calibration curves were prepared for all the four bacterial strains as follows: After the 12-14 hrs cultivation of the bacteria, cells were harvested by centrifugation at 5,000 rpm for 5 min to remove the culture media. PBS buffer was then added and mixed well to achieve the concentration of approximately 8 × 10^8^ cells/mL as the calibration solution I (CS-I) and the optical density at the wavelength of 600 nm (OD_600_) was measured by Nanodrop 2000C (Thermo Fisher Scientific). The cell density was estimated by assuming that the OD_600_ of 1.0 equals 8 × 10^8^ cells/mL and that the cell density is proportional to the OD_600_ values within the range of 0.1-1.0. The CS-I was then progressively diluted with PBS to achieve five calibration solutions with the cell concentration range from 8 × 10^8^ to 8 × 10^4^ cells/mL. The cell densities of the calibration solutions were calculated by the cell density of CS-I times the respective dilution rates. The five calibration solutions were detected by qPCR in triplicates based on the same method described in the section of *DNA quantification by qPCR* in main content. The average PCR cycles numbers (C_T_) for calibration solutions and the estimated cell densities were used to prepare the calibration curves of C_T_ - log_10_ cell concentrations. The linear regression coefficients (*R^2^*) of the calibration curves range from 0.994 to 0.999 and PCR efficiencies are 90.2%-95.7% for all the four bacteria strains tested in this study (shown in Figure S3).

**Figure S2**. The qPCR amplification curves.

**Figure S3**. The qPCR calibration curves for four different bacteria with *R^2^* and percentage PCR efficiency (E, E = 10 ^(-1/slope)^ – 1 where the slope is derived from the linear fitted line of the standard curve).

**Detailed simulation methods**

The flow field within the cathodic chamber was first simulated by *laminar bubbly-flow* module, which calculates the fluid movement induced by the generation and venting of H_2_ during electrolysis. The convective and diffusive OH^-^ transport under the calculated flow field was then modeled by *transport of dilute species* module. Free tetrahedral mesh calibrated for fluid dynamics was used with predefined element size, which was set as fine for all boundary surfaces and as normal for the rest of the geometry.

For flow field simulation, *laminar bubbly-flow* module uses Euler-Euler model to solve two-phase flow macroscopically by tracking phase averaged parameters and volume fraction of each phase [2]. Molar influx of H_2_ gas at the cathodic electrode surface was theoretically half of hydroxide ion generation rate $\text{R}_{\text{in}}^{\text{cat}}$ at the cathode surface, calculated by [3]:

$R_{in}^{cat}=\frac{i}{nFA}$ (1)

where $i$ is the supplied current at 40 mA, *n* is the number of electrons used to generate a hydroxide ion, which is 1, *F* is Faraday’s constant, and *A* is the surface area. The bubble diameter was set at 100 µm which is a typical size reported by Matsushima *et al.*[4, 5]

For *transport of dilute species* interface, OH^-^ generation from the cathodic electrode surface was represented by a uniform inward flux of $\text{R}_{\text{in}}^{\text{cat}}$, calculated by Equation (1) at 1.66 × 10^-3^ mol/(s·m^2^). Simultaneously, in the anodic chamber with 50 mM Na_2_SO_4_ buffer solution, H^+^ ions were produced from the anode surface at the same rate as OH^-^ generation, and cations were forced across the cation exchange membrane. It was assumed that sodium ions were the dominant species transported across the membrane due to their concentration dominance over protons, until sodium ions were transferred down to a concentration comparable to the proton; at this point protons are the preferred ions for membrane transport due to their smaller size. For the cathodic chamber, the influx of H^+^ was considered as the sink of OH^-^ and the contribution of water dissociation was negligible to mass transfer through the membrane [6-8].Therefore, the flux of hydroxide ions at the membrane, $\text{R}_{\text{in}}^{\text{mem}}$, was approximated as a step function:

$R_{in}^{mem}= \left\{\begin{matrix} 0 t<t_{c} \\ -R_{in}^{cat} t\geq t_{c} \end{matrix}\right.$ (2)

where *t* is time and *t_c_* is the critical time when protons become favored for cross membrane transport. The value of *t_c_* was approximated by the time of complete consumption of sodium ion in the anodic chamber. The initial pH was set at 7.5. The time-dependent concentration profile of OH^-^ was analyzed with the diffusion coefficient of OH^-^ in water set at 5×10^-5^ cm^2^/s [9]. From the simulated hydroxide ion concentrations, the transient pH profiles of the cut plane across the electrode and the membrane were generated, while the bulk solution pH was estimated from the volume average of [OH^-^].

**The ECL effects on DNA damage and PCR inhibition**

In order to further understand the decrease of the DNA extraction efficiency with longer ECL durations, the effects of ECL on DNA damage was first investigated. The extracted DNA was used to conduct the ECL experiments instead of bacterial cells. And the DNA samples were collected from *E. coli* cells in 50 mM Na_2_SO_4_ (~10^8^ cells/mL) after 5 min of bead beating in a pre-loaded bead tube (S0205-50, GeneRite, USA) followed by 10 min of centrifuge at 10,000g to remove cell debris. Then the extracted DNA samples were injected into the cathodic chamber and subjected to ECL with different durations from 30 s-30 min. To exclude any potential PCR inhibition effects, the cathodic effluents were purified by a commercial DNA extraction kit (PureLink® Genomic DNA Mini Kit, Invitrogen by Thermo Fisher Scientific, USA) and then measured by qPCR. The DNA concentrations in all the samples were estimated using the calibration curve of Ct-Log C for *E. coli* shown in Figure S3. Then the percentages of DNA loss compared to the initial DNA sample in function of ECL durations are calculated and shown in Figure S4. Approximately 60% less DNA than in the initial sample was detected after 30 min of ECL. This confirms the contribution of DNA damage to the decrease in DNA extraction efficiency, shown in Figure 2 in the main context.

On the other hand, to investigate the influence of PCR inhibition, 50 mM Na_2_SO_4_  was added into both anodic and cathodic chambers, and the electrolyzed cathodic solutions (ECS) were collected after ECL durations of 30 s-30 min. The extracted DNA samples were prepared in the same way as in the DNA damage test above. Then the extracted DNA samples were mixed with different ECS in a ratio of 1:1. All the mixture and the initial DNA samples without adding any ECS were detected by qPCR and qPCR C_T_ values are shown in Figure S4. There was no significant difference observed between the DNA samples mixed with different ECS and the initial DNA sample. It suggests that there were barely any PCR inhibitors generated in the ECS, which could contribute to the decrease in the DNA extraction efficiency. Additionally, all the PCR assays containing ECL samples with different durations were measured as a pH range of 8.4-8.7 (*vide supra*). So, the increased pH in the ECS with different ECL durations should not have an inhibition effect on qPCR detection, either. Therefore, it suggests that the decreased DNA extraction efficiencies with longer ECL durations were predominately resulted by DNA damage during ECL process, *e.g.* the local high pH generated at cathode.

**Figure S4**. Effects of ECL on DNA damage.

**Figure S5**. Effects of electrolyzed cathodic solution (ECS) with varied ECL durations on qPCR detection. The extracted DNA samples in 50 mM Na_2_SO_4_ was added in different ECS with a ratio of 1:1.

**Figure S6**. The cell concentrations of 4 different bacteria in control and electrochemical lysed samples with the measurement by fluorescence image counting.


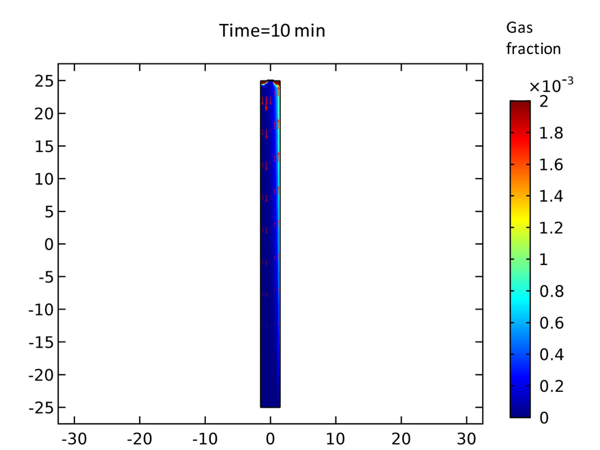


**Figure S7**. Simulated steady-state flow field of the vertical mid-plane across the electrode and the membrane. The gas fraction and velocity field shown in the plot rapidly reached steady-state within 0.1 s, the shortest time step in the simulation. The color surface represents the volume fraction of gas phase. In the superimposed 2D arrow plot of velocity field, it is observed that upward fluid momentum close to the electrode surface (the right edge) was induced by gas motion, and that downward motion on the other side was driven by mass conservation. The fluid in the upper volume was notably accelerated and would boost convective transport of OH^-^ ions.


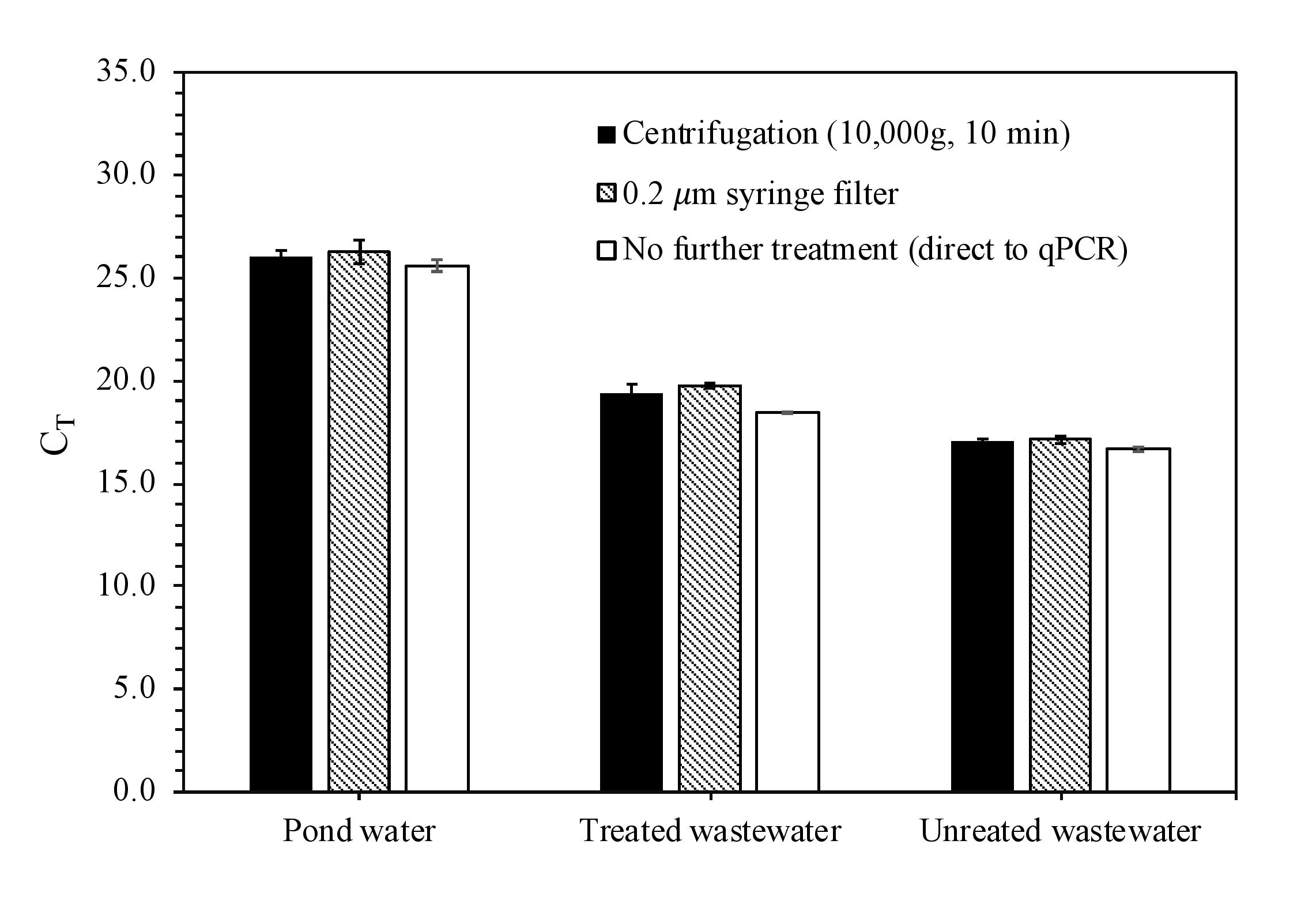


**Figure S8**. The qPCR C_T_ values of total bacteria in environmental water samples with different post-treatment following the optimized ECL reactions (1 min, 10 min and 15 min for pond water, treated and untreated latrine wastewater, respectively).

**References for Supporting Information**

[1] U.S. Environmental Protection Agency. Method B: Bacteroidales in Water by TaqMan® Quantitative Polymerase Chain Reaction (qPCR) Assay, 2010.

[2] E. A. Vera, J. R. Ruiz, In Comparison Between Turbulent and Laminar Bubbly-Flow for Modeling H_2_/H_2_O Separation, COMSOL Conference, Milan, 2012.

[3] A. J. Bard, L. R. Faulkner, J. Leddy, C. G. Zoski, Electrochemical methods: fundamentals and applications. Wiley New York 1980.

[4] H. Matsushima, Y. Fukunaka, K. Kuribayashi, Water electrolysis under microgravity: Part II. Description of gas bubble evolution phenomena. Electrochimica Acta, 51 (2006) 4190-4198.

[5] H. Matsushima, D. Kiuchi, Y. Fukunaka, Kuribayashi, K., Single bubble growth during water electrolysis under microgravity. Electrochemistry Communications, 11 (2009) 1721-1723.

[6] R. Simons, Strong electric field effects on proton transfer between membrane-bound amines and water. Nature, 280 (1979) 824.

[7] J. J. Krol, M. Wessling, H. Strathmann, Concentration polarization with monopolar ion exchange membranes: current–voltage curves and water dissociation. Journal of Membrane Science, 162 (1999) 145-154.

[8] Y. Tanaka, S.-H. Moon, V. V. Nikonenko, T. Xu, Ion-exchange membranes. International Journal of Chemical Engineering, 2012.

[9] S. H. Lee, J. C. Rasaiah, Proton transfer and the mobilities of the H^+^ and OH^−^ ions from studies of a dissociating model for water. The Journal of chemical physics, 135 (2011) 124505.
